# Unilateral opaque chest radiograph in paediatrics: A case series

**DOI:** 10.4102/sajr.v25i1.2164

**Published:** 2021-08-31

**Authors:** Tengku A. Raja Mamat, Khairil A. Sayuti, Chandran Nadarajan, Mohd R. Mohd Zain

**Affiliations:** 1 Department of Radiology, School of Medical Sciences, University Sains Malaysia, Kubang Kerian, Kelantan, Malaysia; 2 Department of Radiology, Hospital University Sains Malaysia, Kubang Keria, Kelantan, Malaysia; 3 Department of Paediatrics, School of Medical Sciences, University Sains Malaysia, Kubang Kerian, Kelantan, Malaysia; 4 Department of Paediatrics, Hospital University Sains Malaysia, Kubang Kerian, Kelantan, Malaysia

**Keywords:** pulmonary underdevelopment, pulmonary agenesis, pulmonary aplasia, pulmonary hypoplasia, congenital disease

## Abstract

Pulmonary underdevelopment is a rare congenital disease which manifests as persistent hemithorax opacification at chest radiography. We present three patients with different types of pulmonary underdevelopment, their imaging features and associated anomalies. Case 1 is a premature neonate with persistent respiratory distress. Further imaging confirmed right pulmonary hypoplasia, associated with a patent foramen ovale, patent ductus arteriosus and vertebral anomalies. Case 2 is a 6-year-old child with corrected anorectal malformation, and recurrent pneumonia. Further imaging confirmed left pulmonary aplasia, associated with an aberrant right subclavian artery and vertebral anomaly. Case 3 is a full term neonate who developed excessive drooling of saliva and respiratory distress. Further imaging confirmed right pulmonary agenesis, associated with an atrial septal defect, patent ductus arteriosus and tracheo-oesophageal fistula. Pulmonary underdevelopment is classified into three types: hypoplasia, aplasia and agenesis. The majority of them have associated anomalies. This condition should be considered a differential diagnosis in paediatric patients with an opaque hemithorax on chest radiography.

## Introduction

Pulmonary underdevelopment is a spectrum of rare malformations, consisting of agenesis, aplasia and hypoplasia.^1^ It is a result of developmental failure of the respiratory system from the foregut. The incidence of pulmonary agenesis and aplasia is between 0.0034% and 0.0097%,^2^ whilst the true prevalence of pulmonary hypoplasia is unknown.^3^ In newborns and children with a unilateral opaque lung and ipsilateral shift of the mediastinum, pulmonary underdevelopment should be included in the differential diagnosis.^2^ We present three cases of pulmonary underdevelopment with a spectrum of imaging findings.

### Case 1

A premature baby boy, born at 28 weeks via breech assisted delivery, was the first twin of a monochorionic diamniotic pair. He was born non-vigorous with poor breathing and weak muscle tone. Intubation was necessary post-delivery because of oxygen desaturation. He was initially treated for respiratory distress syndrome and later complicated with pseudomonas aeruginosa septicaemia.

**FIGURE 1:**
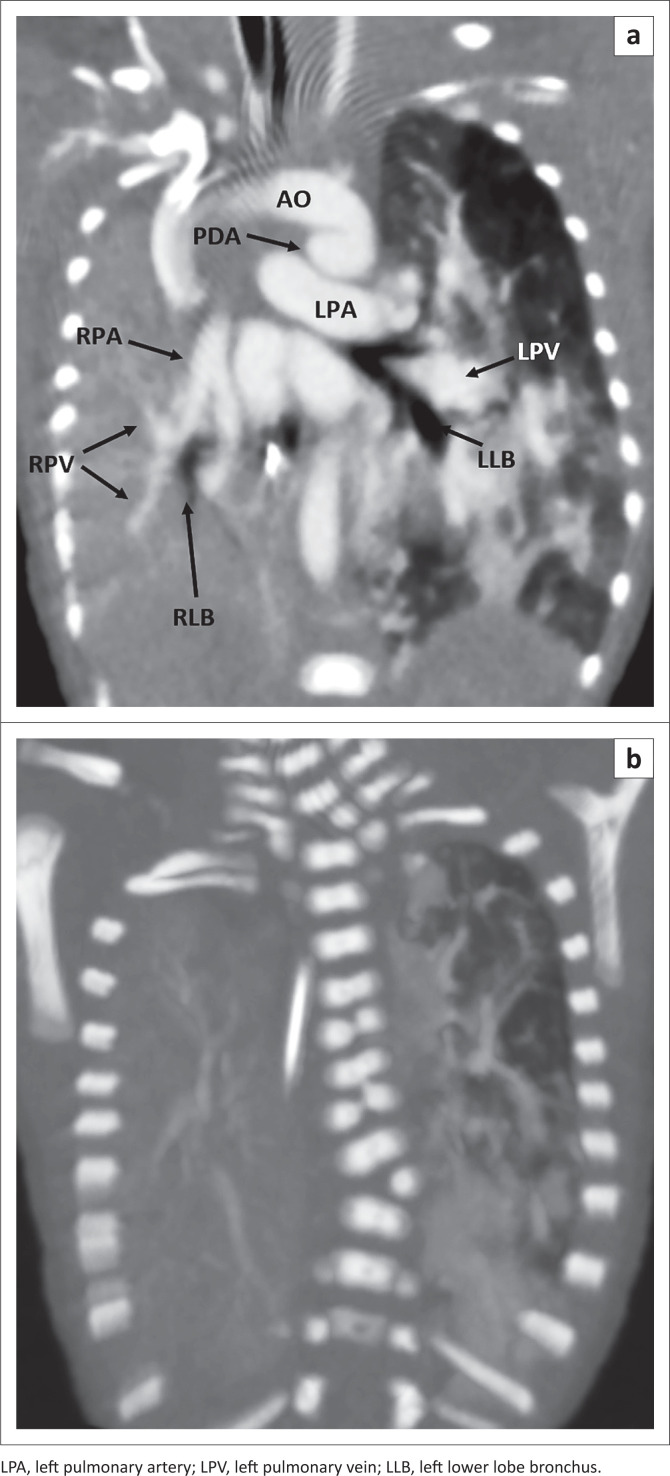
Computed tomography angiography thorax (A) demonstrates hypoplastic right lung, right lower lobe bronchi (RLB), right pulmonary artery (RPA) and veins (RPV). The right hemithorax volume is reduced with ipsilateral mediastinal shift. Large patent ductus arteriosus (PDA) connecting with the aorta (AO). Posterior CT imaging (B) demonstrates left hemivertebrae at C7/T1 and T8/T9, butterfly vertebrae of T5 and T7, and left C7/T1 cervical rib.

Serial chest radiographs (CXR) showed persistent right homogeneous opacity. Echocardiography demonstrated a patent foramen ovale (PFO), a large patent ductus arteriosus (PDA) and mild tricuspid regurgitation. Computed tomography angiography (CTA) of the thorax (Figure 1) revealed a small right hemithorax volume with a hypoplastic right pulmonary artery (RPA) and vein as well as the right segmental bronchi consistent with pulmonary hypoplasia. PFO and a large PDA were present. At thoracic spine radiography, there was levoscoliosis with lateral left hemivertebrae at C7/T1 and T8/T9, butterfly vertebrae of T5 and T7, and a left C7/T1 cervical rib (Figure 1).

### Case 2

A 6-year-old girl underwent an anorectal malformation correction at another hospital when she was very young. She also presented with recurrent episodes of pneumonia. In view of persistent homogeneous opacification of the left hemithorax on CXR, she underwent CT thorax at 4 months of age which revealed a congenital anomaly of the left lung.

**FIGURE 2:**
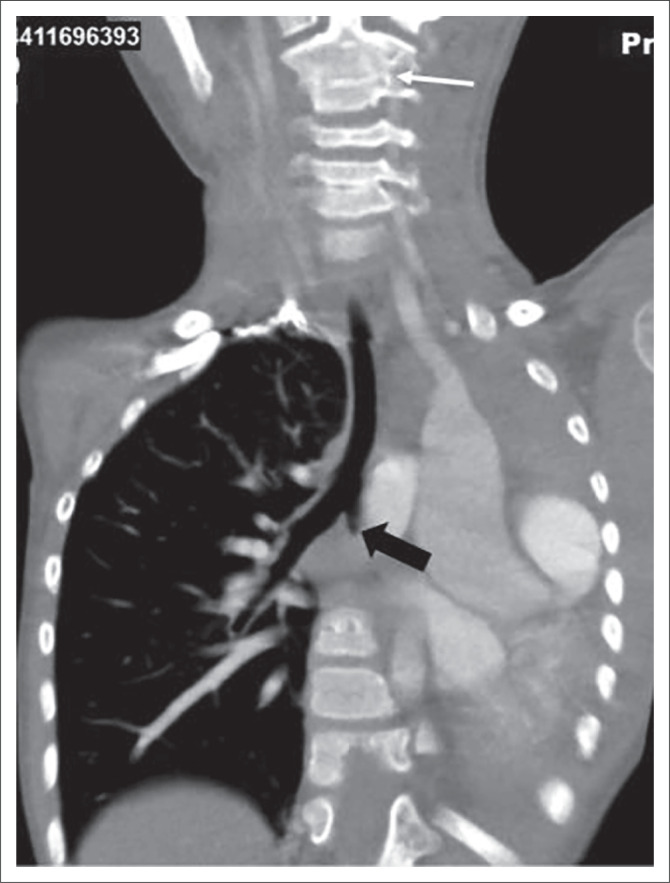
Coronal Computed tomography angiography thorax demonstrates absence of the left lung and pulmonary vessels. The LMB shows abrupt termination after the carina (thick black arrow). The mediastinum is completely displaced to the left side. There is associated fusion of the C2–C3 vertebrae (thin white arrow).

She was referred to our hospital with gradually increasing exertional dyspnoea, which restricted her physical activities. CTA thorax (Figure 2) revealed reduced left hemithoracic volume, a hyperexpanded right lung and total mediastinal shift to the left. The left lung, pulmonary artery and vein were completely absent. The left main bronchus (LMB) showed abrupt termination, about 0.5 cm from the carina. There was an aberrant right subclavian artery with a retro-oesophageal course at the T4 level. The C2/C3 vertebral bodies were fused (Figure 2).

### Case 3

**FIGURE 3:**
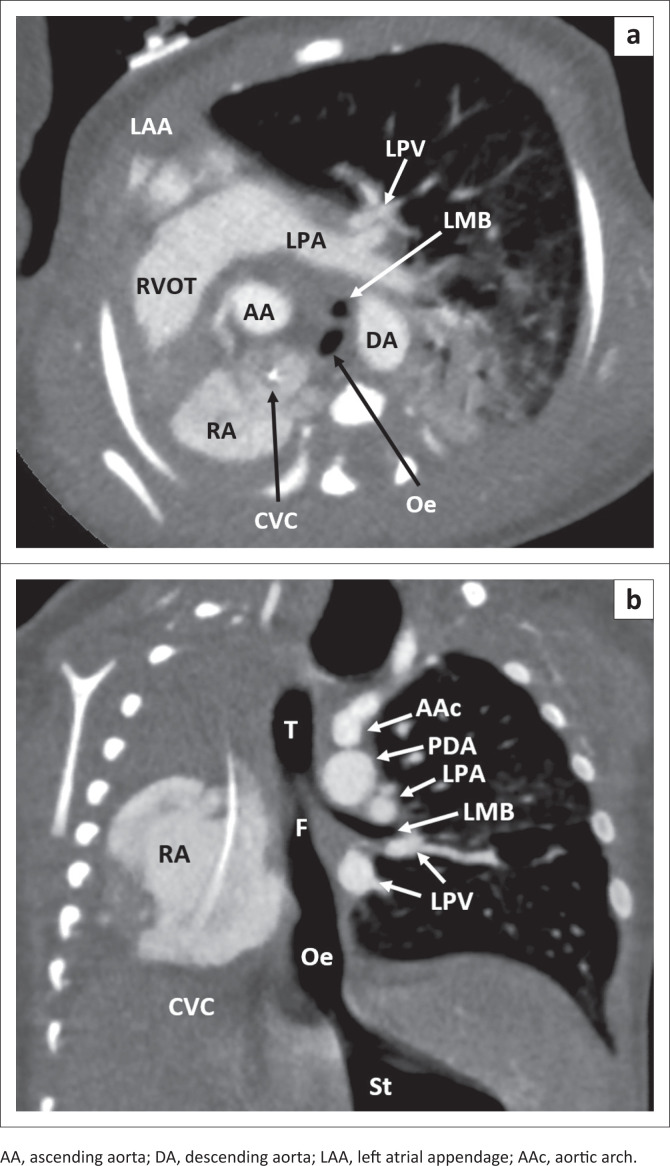
Computed tomography angiography thorax in the axial (A) and oblique coronal (B) planes reveals absence of the right lung, bronchus and pulmonary vessels with ipsilateral mediastinal shift. The left pulmonary artery (LPA) and veins (LPV) are present with a hyperinflated left lung. The trachea (T) divides into the left main bronchus (LMB) and communicates with the lower oesophagus (Oe) via a fistula (F) consistent with a tracheo-oesophageal fistula. The patent ductus arteriosus (PDA) is large. The tip of the central venous catheter (CVC) is in the right atrium (RA).

A full term baby boy was born with good Apgar scores. Antenatal ultrasound had revealed polyhydramnios. Several hours after delivery, the baby developed excessive drooling of saliva and increasing respiratory distress, requiring intubation. The right breath sounds were absent with limited advancement of the feeding tube. CXR revealed complete opacity of the right hemithorax, ipsilateral mediastinal shift, contralateral lung hyperinflation and looping of the feeding tube at mid upper thoracic level. Echocardiography demonstrated dextrocardia, an atrial septal defect (ASD) and a PDA. CTA thorax (Figure 3) revealed total absence of the right lung, right main bronchus and right pulmonary vessels in keeping with pulmonary agenesis. The upper oesophagus was atretic.

The lower oesophagus communicated directly with the carina in keeping with a tracheo-oesophageal fistula. The main pulmonary artery was 1.5 times larger than the ascending aorta suggestive of pulmonary hypertension (Figure 3). The baby underwent thoracostomy and the fistula below the LMB was ligated, followed by gastrostomy tube insertion.

## Outcomes

Both cases 1 and 3 succumbed at very young ages. Case 1 had recurrent oxygen desaturation episodes and passed away at 32 weeks of age. Case 3 died 1 week post-surgery because of a nosocomial lung infection and deteriorated cardiac function.

Case 2 was discharged home with a salbutamol metered-dose inhaler (MDI) as required. She had occasional rapid breathing and wheezing during strenuous physical activities, and also when experiencing an upper respiratory tract infection. The symptoms resolved with the salbutamol MDI. She displayed failure to thrive despite having a good appetite.

## Discussion

Pulmonary underdevelopment is classified into three groups.^3^ It was originally classified by Schneider and Schawatbe^4^ and has been modified by Boyden.^5^ Type 1 is called agenesis, and it refers to complete absence of the lung, bronchus and vascular supply to the affected side. Type 2 is aplasia, where the pulmonary parenchyma and pulmonary vessels are completely absent with the presence of a rudimentary bronchus. Type 3 is named hypoplasia, which is characterised by the presence of a bronchus and rudimentary lung, with decreased size and number of the airways, alveoli, and pulmonary vasculature.^6^ In this case series, case 1 would be classified as hypoplasia, case 2 as aplasia, and case 3 as agenesis.

It is suggested that pulmonary agenesis and aplasia may be considered as one entity clinically and developmentally.^1^ Pulmonary agenesis was first discovered by De Pozze in 1673 during an autopsy of an adult woman.^7^ It was first described as a clinical entity by Klebs in 1874, when he described it as a ‘missing lung’.^8^ The exact aetiology of pulmonary agenesis is unknown.^9^ It is hypothesised that pulmonary agenesis is caused by an abnormal blood supply in the dorsal arch during the 4th week of gestation.^6^ Another suggested hypothesis is that the disease is caused by the disruption of the epithelial-mesenchymal cross-talk, which is important in pulmonary differentiation.^10^ Some of the suggested factors include genetic, viral agents, and vitamin A deficiency during pregnancy.^2^

Pulmonary agenesis is generally sporadic, with only a few reported cases of autosomal recessive inheritance. It has no gender predilection, and it affects both lungs equally.^11^ It has a wide range of clinical features ranging from asymptomatic to variable respiratory symptoms and recurrent lung infection. The onset of the symptoms is variable. The symptoms may start in neonates or later during childhood or even in adulthood;^12^ the oldest reported case is a 72-year-old.^9^ More than 50% of cases have associated abnormalities involving the cardiovascular, gastrointestinal, genitourinary and skeletal systems^6^ as well as ipsilateral facial asymmetry.^8^ About 50% of babies with pulmonary agenesis are either stillborn or die in the 1st month of life.^13^ In this case series, cardiovascular and gastrointestinal anomalies were seen in cases 2 and 3, whilst vertebral anomalies were present in case 2.

On the other hand, pulmonary hypoplasia can be thought as either primary or secondary. Primary pulmonary hypoplasia is less common, and it is when the cause cannot be identified.^6^ An embryological defect of the lung, or *in-vitro* lung injury are some of the suggested aetiologies.^3^ Secondary pulmonary hypoplasia is because of conditions that limit the thoracic space for lung development, which can either be extrathoracic or intrathoracic. Congenital diaphragmatic hernia is the most common intrathoracic cause. Severe oligohydromnios is the most common extrathoracic cause, which can be either secondary to genitourinary abnormalities or prolonged rupture of membranes.^6^ Other causes include congenital heart diseases, neuromuscular disorders, thoracic cage abnormalities (Jeune syndrome, asphyxiating thoracic dystrophy), genitourinary tract anomalies and Scimitar syndrome. Patients with pulmonary hypoplasia typically present with early respiratory distress after birth, or with life-threatening symptoms such as cyanosis and hypoxia in childhood.^3^ This is similar to case 1 where the patient was found to have associated cardiovascular and vertebral anomalies, and died at 32 weeks after recurrent episodes of oxygen desaturation.

The aetiologies of unilateral thorax opacification vary according to age. The five most common aetiologies are large pleural effusion, obstruction of the main bronchus, pneumonia, intrathoracic tumours and pulmonary underdevelopment. In neonates, congenital diaphragmatic hernia, congenital large hyperlucent lobe and congenital thoracic malformation should also be considered as the differential diagnoses.^14^

The position of the mediastinum provides important clues to the underlying conditions. Contralateral mediastinal shift is seen in space occupying entities like intrathoracic tumours and large pleural effusion.^14^ In neonates, the pleural effusion is usually chylous. If the density of the involved hemithorax is heterogeneous, the diagnosis of volume-occupying congenital malformations such as cystic pulmonary airway malformation, congenital diaphragmatic hernia and pulmonary sequestration should be entertained.^15^ Ipsilateral mediastinal shift is seen in conditions where the lung volume is reduced or absent, such as lung collapse and pulmonary underdevelopment. Apart from the ipsilateral mediastinal displacement, additional signs for reduced lung volume include crowding of the ribs and elevated ipsilateral hemidiaphragm.^14^ The common causes of unilateral lung collapse in neonates include a malpositioned endotracheal tube and post extubation collapse. In conditions where the lung volume is preserved, such as pneumonia, the mediastinal structures typically remain at a central position.^14^

In patients with reduced or absent breath sounds, a decrease or absence of movement of the unilateral chest wall and an opaque hemithorax on chest radiograph, the diagnosis of pulmonary agenesis should be considered.^12^ In pulmonary hypoplasia, a typical finding is ipsilateral mediastinal shift which is accentuated on inspiration because of increased compensatory hyperinflation of the contralateral lung.^3^ Bronchoscopy and bronchography may be necessary to confirm the diagnosis, whilst angiography is also required in some cases.^9^ In all our patients, CXRs showed homogeneous opacity of the involved hemi-thoraces.

The prognosis of pulmonary underdevelopment is largely dependent upon the remaining functional lung parenchyma, as well as the presence and severity of the associated abnormalities.^9^ One of the complications of the disease is pulmonary fibrosis as a result of recurrent chest infection.^12^ Right-sided pulmonary underdevelopment has been reported to have a worse prognosis because of the greater distortion of the heart and mediastinum,^11^ and compression of the tracheo-bronchial tree.^2^ In this case series, cases 1 and 3 died at very young ages. These patients had right-sided involvement of the disease, and both of them had associated cardiovascular anomalies. Case 2 had recurrent pneumonia and occasional exertional dyspnoea on vigorous physical activities, which resolved with medication.

## Conclusion

Pulmonary underdevelopment has a wide range of clinical presentations and can occur at any age. The severity of the disease ranges from hypoplasia to agenesis, and there may be variable degrees of associated anomalies. Even though it is rare, it should be considered as one of the differential diagnoses, especially in patients with an opaque unilateral hemithorax on plain film radiography. An early diagnosis is important for early initiation of the treatment, thereby preventing possible complications.
